# Effect of urine pH changed by dietary intervention on uric acid clearance mechanism of pH-dependent excretion of urinary uric acid

**DOI:** 10.1186/1475-2891-11-39

**Published:** 2012-06-07

**Authors:** Aya Kanbara, Yoshisuke Miura, Hideyuki Hyogo, Kazuaki Chayama, Issei Seyama

**Affiliations:** 1 Department of Nutrition and Health Promotion, Faculty for Human Development, Hiroshima Jogakuin University, 4-13-1 Ushita-higashi Higashi-ku, Hiroshima, 732-0063, Japan; 2 Department of Medicine and Molecular Sciences, Graduate School of Biomedical Sciences, Hiroshima University, 1-2-3 Kasumi, Minami-ku, Hiroshima, 734-8551, Japan

**Keywords:** Hyperuricemia, Gout, Dietary intervention, Acid–base

## Abstract

**Background:**

The finding reported in a previous paper - alkalization of urine facilitates uric acid excretion - is contradictory to what one might expect to occur: because food materials for the alkalization of urine contain fewer purine bodies than those for acidification, less uric acid in alkaline urine should have been excreted than in acid urine. To make clear what component of uric acid excretion mechanisms is responsible for this unexpected finding, we simultaneously collected data for the concentration of both creatinine and uric acid in serum as well as in urine, in order to calculate both uric acid and creatinine clearances.

**Methods:**

Within the framework of the Japanese government’s health promotion program, we made recipes which consisted of protein-rich and less vegetable-fruit food materials for H ^+^ -load (acidic diet) and others composed of less protein and more vegetable-fruit rich food materials (alkaline diet). This is a crossover study within some limitations. Healthy female students, who had no medical problems at the regular physical examination provided by the university, were enrolled in this consecutive 5-day study for each test. From whole-day collected urine, total volume, pH, organic acid, creatinine, uric acid, titratable acid and all cations (Na^+^,K^+^,Ca^2+^,Mg^2+^,NH_4_^+^) and anions (Cl^−^,SO_4_^2−^,PO_4_^−^) necessary for the estimation of acid–base balance were measured. In the early morning before breakfast of the 1st, 3rd and 5th experimental day, we sampled 5 mL of blood to estimate the creatinine and uric acid concentration in serum.

**Results and discussion:**

Urine pH reached a steady state 3 days after switching from ordinary daily diets to specified regimens. The amount of acid generated ([SO_4_^2−^] + organic acid − gut alkali)was linearly related with the excretion of acid (titratable acid + [NH_4_^+^] − [HCO_3_^−^]), indicating that H ^+^ in urine is generated by the metabolic degradation of food materials. Uric acid and excreted urine pH retained a linear relationship, as reported previously. Among the five factors which are associated with calculating clearances for both uric acid and creatinine, we identified a conspicuous difference between acidic and alkaline diets in the uric acid concentration in serum as well as in urine; uric acid in the serum was higher in the acidic group than in the alkaline group, while uric acid in the urine in the acidic group was lower than that in the alkaline group. These changes of uric acid in acidic urine and in serum were reflected in the reduction of its clearance. From these observations, it is considered that uric acid may be reabsorbed more actively in acidic urine than in alkaline urine.

**Conclusion:**

We conclude that alkalization of urine by eating nutritionally well-designed alkaline -prone food is effective for removing uric acid from the body.

## Introduction

In a previous paper (2010) [1], we reported a potential utilization of dietary intervention for reducing hyperuricemia :diet-induced alkaline urine excretes more uric acid than acidic urine. Taking into account the observations by Griesch and Zöllner (1975) [2] and Clifford, Riumallo, Young and Scrimshaw (1976) [3] that purine bodies loading through the diet induce a proportional increase in serum uric acid concentration, the more purine bodies were loaded in the acidic diet, the more uric acid should have been excreted in the urine. However, what we observed in a previous study was the exact opposite. One way to resolve this issue is to measure simultaneously the concentrations of both uric acid and creatinine in serum as well as in urine, to calculate uric acid and creatinine clearances. By so doing, one can obtain information on how uric acid is handled along the renal tubule after the filtration, in such a way that uric acid reabsorption proceeds in the proportion of decrease in urine pH.

In this report, we provide conclusive evidence for the efficiency of dietary intervention for the prevention of hyperuricemia by showing that acidic urine causes less uric acid to be excreted from the body than alkaline urine does.

## Methods

### Subjects

Eighteen female university students (five students for 2010 and thirteen students for 2011), 21-22 years old, participated in this study. The ethics committee at Hiroshima Jogakuin University approved the study protocol. All subjects signed informed consent documents. Although four out of the eighteen participants during the acidic diet period and three during the alkaline diet period were obliged to discontinue the project due to menstruation, the rest continued to participate in this project. Thus, this became a crossover study in which subjects were not completely overlapped. The health condition of all participants was monitored by measuring body weight, changes in which were very limited during the experiment periods (within less than 1% compared to body weight at the beginning).

### Diet

Values for protein, energy and purine contents were extracted from the available data in the standard tables of food composition in Japan 5th revised and enlarged edition issued by the Ministry of Education, Culture, Sports, Science and Technology Japan for all diets ingested by the eighteen subjects. Resultant calculation of the contents of whole protein and purine bodies in food materials yielded 56.2 g/d in the alkaline diet and 95.4 g/d in the acidic diet for protein, and 351 mg/d in the alkaline diet and 494 mg/d in the acidic diet for purine bodies, respectively. Amino acids in the diet which can generate an acid in the catabolic process, such as arginine, lysine, 1/2 histidine, methionine and cystein (in mmol), were present in 57 mmol/d in the alkaline diet and 124 mmol/d in the acidic diet. Each diet period lasted five days. During each five-day period, diets made by different recipes but using the same compositions of natural food materials were served. Foods used during the experimental period in 2011 for both the acidic and alkaline diets are listed in the Appendix as representative data. Subjects had free access to mineral water. The first and second diet periods were separated by one month.

### Collection of specimens

Twenty-four-hour urine specimens were collected in bottles and stored in a refrigerator. Volume, pH, titratable acid, organic acid and creatinine were measured in a sample from urine collected the day before the measurement. A four mL urine sample for each experimental day for every person was stored in a deep freezer for later ion analysis. Blood samples were collected in the early morning before breakfast on the 1st, 3rd and 5th experimental days.

### Analytical methods

According to Lennon, Lemann, Jr. and Litzow (1966) [4], the production of endogenous acid is determined by the sum of 1) the oxidation of sulfate in the sulfur-containing amino acids, 2) the endogenous formation of unmetabolized organic acids, and 3) the net gastrointestinal absorption of alkali produced by the oxidation of organic cations and anions. Using the simplified method proposed by Oh (1989) [5], data necessary for the net gastrointestinal absorption of alkali (Na^+^,K^+^,Ca^2+^,Mg^2+^,NH_4_^+^,Cl^−^,PO_4_^−^,SO_4_^2–^) (mEq) were obtained using HPLC. The details of methods employed may be consulted in a previous paper [1]. The excretion of endogenous acid consists of titratable acid, ammonium ion and bicarbonate ion [4]. Titratable acid was estimated as the amount of 0.1 mol NaOH necessary to titrate back to pH 7.4 from urine pH. Organic acid salts were measured by the Van Slyke and Palmer method (1920) [6]. The organic acid salt measured was corrected for titration of creatinine which was determined by the Folin method. Bicarbonate concentration ([HCO_3_^−^) was calculated using the Henderson-Hasselbach equation for which the solubility coefficient of carbon dioxide was taken as 0.0309 mmol/mmHg·L and P_Ka_ and P_CO2_ were assumed to be 6.10 and 40 mmHg, respectively. Urine pH was measured at 37 °C with a pH meter. Uric acid was measured by the conventional uricase-peroxidase method, using an autoanalyzer.

### Statistical analysis

Data were presented as mean ± SD. The student *t* test was used to test the significance of changes in measured parameters between the acidic and alkaline periods. Differences were assumed to be significant when p < 0.05.

## Results

### Relationship between acid generation in the body and acid excretion in urine

In order to confirm that the proper loading of acid had been achieved, we measured several factors related to the endogenous fixed acid production and urinary acid excretion. We listed urinary ammonium, phosphate, titratable acid, bicarbonate and sulfate together with urinary pH as typical representatives in Table 1 and cationic and anionic ion species in relation to the net gastrointestinal absorption of alkali in Table 2. The significant difference in urine [SO4 2−] between the acidic and alkaline diets was associated with the amount of sulfur-containing amino acid in foods of 28.8 mmol/d in the acidic and 14.5 mmol/d in the alkaline diets. Urinary ammonium, phosphate and sulfate were inversely related to the time course of urinary pH. On the intake of the alkaline diet, these values were significantly lower than those in the acidic diet (Table 1). It is interesting that gut alkali {([Na^+^ + [K^+^ + [Ca^2+^ + [Mg^2+^)-([Cl^−^ + 1.8[PO_4_^−^)} in the acidic diet yielded much smaller value than in the alkaline diet (Table 2, bottom line). Because the generation of acid is calculated by ([SO_4_^2−^ + organic acid − gut alkali) [4], gut alkali in the acidic diet contributes to the small decrease of acidity compared with that in the alkaline diet.

**Table 1:** Comparison of estimated urinary excretion of ions associated with the acid–base balance

	**alkaline diet (n=30)**	**acidic diet (n=27)**	**p**
urine volume (L/d)	1.37 ± 0.35	1.37 ± 0.65	NS
pH	6.51 ± 0.34	5.92 ± 0.28	<0.01
ammonium (mmol/d)	24.11 ± 12.78	52.13 ± 13.27	<0.01
phosphate (mmol/d)	24.64 ± 10.37	35.76 ± 14.69	<0.01
sulfate (mmol/d)	9.94 ± 3.25	21.51 ± 6.21	<0.01
titratable acid (mEq/d)	8.7 ± 3.6	25.6 ± 4.1	<0.01
urinary bicarbonate (mEq/d)	4.7 ± 2.3	0.8 ± 0.4	<0.01
uric acid (mg/d)	413.4 ± 81.7	302.8 ± 134.7	<0.01

**Table 2:** Comparison of gut alkali ions excretion in acidic diet with those in alkaline diet

	**alkaline diet**		**acidic diet**		p (between *and**)
	1st day	3 ~ 5 day*	1st day	3 ~ 5 day**	
	(n = 13)	(n = 30)	(n = 14)	(n = 27)	
Na^+^(mEq/d)	124.4 ± 56.2	94.8 ± 37.1	134.4 ± 58.3	108.6 ± 46.5	NS
K^+^(mEq/d)	63.4 ± 38.0	66.7 ± 19.6	55.6 ± 30.0	34.6 ± 11.5	<0.05
Mg^2+^(mEq/d)	4.9 ± 3.4	5.8 ± 4.2	6.8 ± 4.7	5.0 ± 4.1	NS
Ca^2+^(mEq/d)	3.3 ± 3.3	1.7 ± 1.7	5.5 ± 4.3	2.6 ± 1.8	NS
CI^–^(mEq/d)	127.2 ± 35.4	72.9 ± 24.9	86.1 ± 35.8	89.1 ± 29.3	NS
PO_4_^2-^(mEq/d)	30.1 ± 12.7	27.6 ± 9.9	42.1 ± 19.3	52.7 ± 15.1	<0.01
gut alkali(mEq/d)	38.5 ± 97.3	66.3 ± 38.1	14.3 ± 88.1	8.7 ± 45.3	<0.01

The calculated total effective fixed acid production correlated closely with renal acid excretion (Tables 1 and 2), indicating that the metabolic degradation of food materials results in H ^+^ appearing in urine.

### Effect of urine pH on uric acid excretion

**Figure 1:**
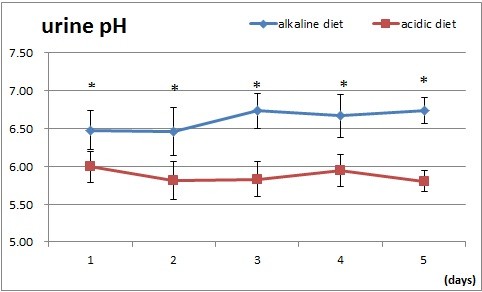
**Effect of acidic (square) and alkaline (diamond) diets on urine pH.** Data are presented as mean ± SD. Asterisks indicate the statistical significance between the two groups (p < 0.002).

**Figure 2:**
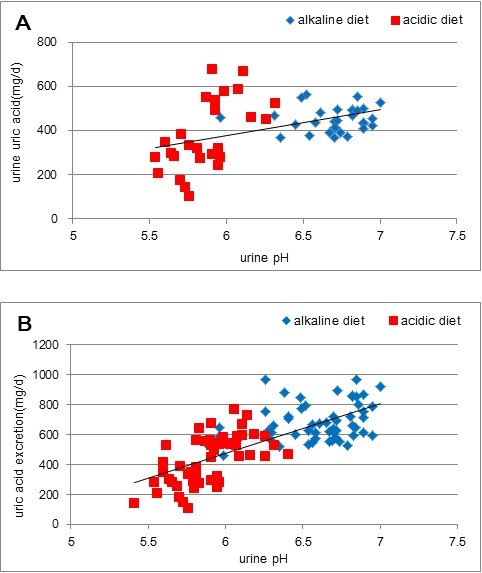
**Relationship between excreted uric acid as expressed in mg uric acid in urine per day and urine pH.** In **A.** Diamonds indicate data for the alkaline diet and squares those for the acidic diet. The equation for the straight line obtained by the least square method is y = 118.9x-335.8 (r2 = 0.2098, n = 57, p < 0.01). In **B.** Corrected relationship between excreted uric acid as expressed in mg uric acid in urine per day and urine pH. Given the loading difference of purine bodies, data for uric acid excreted in alkaline urine were multiplied by correction factors, as described in the text. The equation for the straight line obtained by the least square method is y = 332.25x-1525.6 (r2 = 0.5406, n = 57, p < 0.01).

It took 3 days to reach a steady level of urine pH of 6.7 in the alkaline diet and pH 5.9 in the acidic diet after switching from an ordinary daily diet to either of the designed diets (Figure 1 and Table 1). When urine pH reached a steady state on the third experimental day, data for the excretion of uric acid in urine on the last three experimental days, as expressed as uric acid excretion in mg per day (mg/d), were plotted against urine pH (Figure 2A). The amount of excreted uric acid increased with the increase in luminal pH, and this general trend is consistent with the finding in our previous paper. However, when these data were examined in detail, two different regression lines could be applied to each group of data for acidic and alkaline diets. Given that the content of purine bodies in the alkaline diet was less than in the acidic diet and uric acid in serum is said to be dependent on the loading of purine bodies [2,3], it is reasonable to deduce that the data for alkaline diet may have been underestimated compared with those for the acidic diet. When one roughly corrects these data for the loading difference in purine bodies by multiplying the data for alkaline urine by the ratio of purine bodies in the acidic diet and that in the alkaline of 1.74 for the year 2010 and of 1.4 for 2011, data appear to be aligned along a single straight regression line, as shown in Figure 2B, indicating, at least in part, that their loading difference may well be responsible for the data scattering.

### Differential effects of urine pH on the clearances for creatinine and for uric acid

We conducted simultaneous measurements of creatinine and uric acid in both the serum and urine in order to confirm which factor was responsible for the pH dependent uric acid excretion. Necessary data were available to calculate both creatinine and uric acid clearances − creatinine and uric acid concentrations in both serum and urine, as shown in Table 3. In this paper, because data for the first experimental day was not under influence of designed diets but of ordinary diets, it is reasonable to use them hereafter as baseline data. We calculated the absolute creatinine clearance and uric acid clearance with a correction of body surface area [7]: the mean value of creatinine clearance in alkaline urine was 133.9 ± 19.7 (n = 19) mL/min/1.73 m^2^ and that in acidic urine 138.8 ± 21.4 (n = 19) mL/min/1.73 m^2^ respectively. Although the difference in dietary protein loading should have produced a fluctuation of blood flow in the kidney [8], which in turn might increase urine volume and affect creatinine clearance, its fluctuation in our case was limited, as seen in Table 2. In spite of the difference in mean values, statistical significance was not recognized (p > 0.1). On the other hand, it is noteworthy that the uric acid clearance with a correction of body surface area only in acidic urine significantly decreased from the baseline value of 10.0 ± 1.8 (n = 14) mL/min/1.73 m^2^ to the mean value on the 3rd and 5th experimental days of 6.0 ± 2.2 (n = 19) mL/min/1.73 m^2^, while that in the alkaline urine remained more or less unchanged in the range between the baseline value of 8.9 ± 2.0 (n = 13) mL/min/1.73 m^2^ and the mean value of the 3rd and 5th experimental days of 8.6 ± 2.3 (n = 19) mL/min/1.73 m^2^. Statistical significance was recognized (p < 0.02) in the difference of mean values on the 3rd and 5th experimental days between acidic and alkaline urine. In the case of uric acid, the mean concentrations of excreted uric acid changed from 37.4 ± 17.1 (n = 14) mg/dL for the acidic diet and 45.3 ± 20.4 (n = 13) mg/dL for the alkaline diet as the baseline value to 25.9 ± 10.0 (n = 19) mg/dL of average value for the 3rd and 5th experimental days in the acidic diet and to 37.0 ± 11.2 (n = 19) mg/dL for the 3rd and 5th experimental days in the alkaline diet, respectively (Table 3). Meanwhile, the mean concentration of uric acid in serum for the baseline value in the acidic diet of 4.3 ± 0.6 mg/dL increased to 4.9 ± 0.6 (n = 19) mg/dL for the 3rd and 5th experimental days, but that in the alkaline diet of 4.2 ± 0.8 remained in the same range of 4.3 ± 0.8 (n = 19) mg/dL for the 3rd and 5th experimental days, respectively (Table 3). These data indicate that the disparity of the serum concentration of uric acid between the two diets at least depends on the difference in purine bodies loading, because, in our experiment, the acidic diet contained more purine body at 494 mg/d than the alkaline diet at 351 mg/d. The data obtained are consistent with previous reports [2,3] that serum uric acid concentration is proportional to dietary loading of purine body. When the uric acid clearance was calculated, the difference in uric acid concentration in both urine and serum between the acidic and alkaline diets showed a strikingly lower uric acid clearance in the acidic diet compared with that in the alkaline diet (Table 3).

**Table 3:** **Effect of diet-induced H**^**+**^**load on factors for both creatinine and uric acid clearances**

	**alkaline diet**	**acidic diet**	**p**
	**(n = 19)**	**(n = 19)**	
serum uric acid (mg/dL)	4.3 ± 0.8	4.9 ± 0.6	<0.01
serum creatinine (mg/dL)	0.58 ± 0.08	0.63 ± 0.06	NS
urine uric acid (mg/dL)	37.0 ± 11.2	25.9 ± 10.0	<0.01
urine creatinine (mg/dL)	78.9 ± 24.3	79.3 ± 18.0	NS
urine volume (mL/day)	1270 ± 436	1429 ± 320	NS
Uric acid clearance (mL/min/1.73 m^2^)	8.6 ± 2.3	6.0 ± 2.2	<0.01
Creatinine clearance (mL/min/1.73 m^2^)	133.9 ± 19.7	138.8 ± 21.4	NS

All numerical data were collected from records during the 3rd and 5th experimental days.

**Figure 3:**
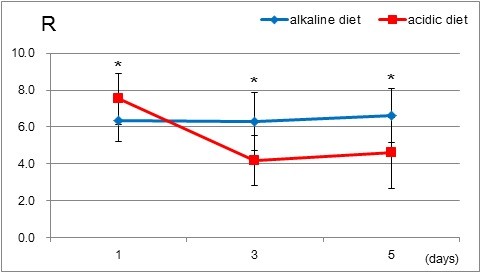
**Change in fractional uric acid excretion, Rs (uric acid clearance/creatinine clearance) were obtained and plotted on each experimental day.** Asterisks indicate the statistical significance between the two groups (p < 0.002).

In the circumstance, as in this study, in which healthy students live sedentary lives, taking nearly ordinary diets, these stable creatinine clearances may be reasonably assumed to be close to the glomerular filtration rate. Thus, to make it easy to resolve what mechanism was at work here, we took the ratio of uric acid clearance to creatinine clearance:fractional uric acid excretion, calling it R where R = ([uric acid in a unit urine] × [creatinine in a unit serum]/[uric acid in a unit serum] × [creatinine in a unit urine]) × 100 (%). While creatinine clearances in both acidic and alkaline diets remain in a similar range, it has been found that uric acid clearance in an acidic diet is suppressed. Thus, it is reasonable to assume that R in an alkaline diet should be larger than in an acidic diet. As shown in Figure 3, that was the case. R in the alkaline diet on both the 3rd and 5th experimental days was consistently greater than that in the acidic diet after the settling down of urine pH to a steady state level.

## Discussion

The most conspicuous result in this study is the provision of additional experimentally proven evidence for the previous study, that uric acid excretion is more favorable in alkaline urine than in acidic urine. The strategy taken here for reconfirming our previous study was to collect data for calculating the clearances for both uric acid and creatinine. As seen in Table 3, among the five parameters, uric acid and creatinine concentrations, both in urine and in serum and urine volume, a difference between alkaline and acidic diets is only detected in the concentration of uric acid in serum and in urine. Both the decrease in uric acid excretion and the increase in serum uric acid concentration in the acidic diet during the 3rd and 5th experimental days made the uric acid clearance significantly lower than that in the alkaline diet. Taking into account that in the physiological condition urate handling involves urate glomerular filtration followed by a complex array of reabsorptive and secretory mechanisms taking place in the proximal tubules, resulting in fractional excretion of urate being 10% [9] of glomerular filtrate, either secretive or reabsorptive function of uric acid may be able to participate in excretion of urate modified by the change in urine pH. Although the relative importance of the reabsorption and secretion mechanisms has not yet been determined in detail due to the complexity of renal urate handling, a dominant factor for controlling urate excretion is considered to be reabsorption, because a hereditary dysfunction of URAT 1(urate/organic anion exchanger)-lack of reabsorption of luminal urate through URAT 1- causes severe hypouricemia [10]. Thus, for the sake of simplicity in this paper modification of renal urate transport by the change in urine pH is tentatively assumed to be confined to the reabsorption mechanism. The findings so far obtained show that uric acid excretion was greater in the alkaline diet than in the acidic diet, indicating that reabsorption ability may be enhanced in an acidic medium. Further confirmation of reabsorption enhancement in an acidic medium is given by the calculation of clearances for both creatinine and uric acid. Resultant creatinine clearances for acidic and alkaline diets were in a similar range, indicating that the glomerular filtration rate in both acidic and alkaline diets remains stable. As shown in Figure 3, Rs in the alkaline diet are consistently larger than in the acidic diet after urine pH has settled down to a steady state level. These findings strongly indicate that, after complicated absorption and secretion processes on both apical and basolateral membranes in the proximal tubule have taken part in uric acid transport after filtration [9-11], uric acid excretes more favorably in alkaline urine than in acidic urine. The molecular identity responsible for pH-dependent uric acid transport is still unclear, but at the moment not quantitative but qualitative characteristics of human organic anion transporter 4 (hOAT4) [12] support our findings, because a main urate transporter in the proximal tubule is claimed to be URAT1 [9].

Since the study was conducted exclusively on female students, experiments of the same kind need to be performed on male students, on older populations and also on people with hyperuricemia at baseline for generalization of dietary intervention. Given the recent dramatic increase in the incidence of gout and hyperuricemia associated with cardiovascular diseases and metabolic syndrome [13], it is highly desirable to introduce a safe and economic way to reverse these trends. The dietary intervention procedure for cardiovascular diseases [14] and diabetes mellitus, including metabolic syndrome [15] and cancer [16], shares with its similar focus on gout and hyperuricemia the recommendation of taking a sufficient amount of alkaline-rich fruits and vegetables. Because people can take a preventive procedure for these diseases without side effects by following this suggestion, we believe that the dietary intervention we are proposing is one of the best choices.

## Appendix

### (alkaline diet)

White rice 100 g, rye bread 70 g, pasta 80 g, starch 20 g, hard tofu 50 g, silken tofu 50 g, pressed tofu 30 g, fried tofu 6 g, okara 40 g, green soybeans 10 g, milk 150 g, carrot 20 g, leaf vegetable 65 g, tomato 120 g, pepper (red & yellow) 30 g, pumpkin 80 g, green onion 15 g, onion 50 g, cucumber 60 g, cabbage 60 g, lettuce 30 g, garlic 5 g, potato 100 g, aroid 45 g, yam 30 g, mushroom 40 g, kiwi 70 g, pineapple 50 g, walnuts 15 g, dried seaweed 1 g, sugar 8 g, honey 20 g, olive oil 5 g, salad oil 10 g, dressing 8 g, butter 10 g, soy saurce 5 g, vinegar 3 g, soup prepared from dried bonito and tangle 180 g, alcohol for cooking 15 g, miso(fermented soybeans paste) 7 g, sauce 20 g, Japanese basil 1 g, sweet cooking rice wine 4 g, salt 0.5 g, pepper 0.06 g, mayonnaise 10 g.

### (acidic diet)

White rice 185 g, roll 75 g, pasta 20.5 g, starch 6 g, pork shoulder 90 g, cero 90 g, chicken breast fillet 30 g, squid 30 g, egg 100 g, processed cheese 20 g, carrot 50 g, broccoli 20 g, snap pea 20 g, asparagus 20 g, green onion 10 g, bamboo sprout 40 g, corn 25 g, onion 50 g, burdock 15 g, sprout 20 g, tomato saurce 15 g, soy source 6 g, salt 0.8 g, sweet cooking rice wine 12 g, alcohol for cooking 20 g, pepper 0.09 g, miso 9 g, consommé 1 g, soup prepared from dried bonito and tangle 170 g, mayonnaise 9 g, butter 3 g, salad oil 6 g, sugar 9 g, strawberry jam 20 g.

## Competing interests

The authors declare that they have no competing interests.

## Authors’ contributions

AK carried out the analysis of all urine contents and the integration of data into the report. YM, HH and KC participated in the design of the study and helped to draft the manuscript. IS conceived of the study, helped to draft the manuscript and participated in analysis and integration of data. All authors read and approved the final manuscript.
